# More Guidelines than states: variations in U.S. lead screening and management guidance and impacts on shareable CDS development

**DOI:** 10.1186/s12889-020-8225-8

**Published:** 2020-01-29

**Authors:** Jeremy J. Michel, Eileen Erinoff, Amy Y. Tsou

**Affiliations:** 1 Department of Biomedical and Health Informatics, The Children’s Hospital of Philadelphia, 2716 South Street, Philadelphia, PA 19146 USA; 2 Department of Pediatrics, Perelman School of Medicine at the University of Pennsylvania, Philadelphia, PA 19146 USA; 3 ECRI Institute Center for Clinical Evidence and Guidelines, Plymouth Meeting, PA 19462 USA; 4 Michael J Crescenz Veterans Affairs Medical Center, Philadelphia, PA 19104 USA

**Keywords:** Lead Screening, Lead poisoning, Clinical decision support, Geographic variation in care

## Abstract

**Background:**

Pediatric lead exposure in the United States (U.S.) remains a preventable public health crisis. Shareable electronic clinical decision support (CDS) could improve lead screening and management. However, discrepancies between federal, state and local recommendations could present significant challenges for implementation.

**Methods:**

We identified publically available guidance on lead screening and management. We extracted definitions for elevated lead and recommendations for screening, follow-up, reporting, and management. We compared thresholds and level of obligation for management actions. Finally, we assessed the feasibility of development of shareable CDS.

**Results:**

We identified 54 guidance sources. States offered different definitions of elevated lead, and recommendations for screening, reporting, follow-up and management. Only 37 of 48 states providing guidance used the Center for Disease Control (CDC) definition for elevated lead. There were 17 distinct management actions. Guidance sources indicated an average of 5.5 management actions, but offered different criteria and levels of obligation for these actions. Despite differences, the recommendations were well-structured, actionable, and encodable, indicating shareable CDS is feasible.

**Conclusion:**

Current variability across guidance poses challenges for clinicians. Developing shareable CDS is feasible and could improve pediatric lead screening and management. Shareable CDS would need to account for local variability in guidance.

## Introduction

Pediatric lead exposure in the United States (U.S.) remains a preventable public health crisis with far-reaching consequences [1, 2]. Even low levels of lead have been associated with learning disabilities and Attention Deficit Hyperactivity Disorder (ADHD) [3]. In 2012, the CDC (along with other organizations) changed the definition for elevated lead from ≥10 μg/dL to ≥5 μg/dL, selecting this reference point based on the based on the 97.5th percentile of the BLL distribution in US children aged 1–5 years [4]. Recent lapses in preventive measures have exposed numerous children to elevated lead, with health consequences yet to be determined [5]. As recently as 2016, the Centers for Disease Control (CDC) estimated that 500,000 children tested positive for elevated lead [6]. Preventing pediatric lead exposure is particularly critical, given the potential for lifelong cognitive and behavioral problems [3, 7, 8].

Detecting and mitigating environmental lead exposure remains the only effective way to protect children. The burden for prevention lies primarily on public health departments, implementation of policies by local organizations, and health care providers responsible for screening [9]. However, adherence to screening requirements remains surprisingly low [10, 11]: roughly 40% of Medicaid-enrolled children do not receive screening, despite being mandated to do so through the Early Periodic Screening Diagnostic and Testing (EPSDT) program [6]. Overall, low income and minority children remain at higher risk of lead exposure [6].

Guideline-based clinical decision support (CDS) interventions represent a promising strategy for improving care, and can improve guideline adherence [12–14]. CDS, at its most basic level, includes any tool or system designed to help clinicians make decisions [15]. Frequent examples include documentation templates, order sets, computerized order entry [15]. Ideally, shareable guideline based CDS could allow organizations to use one of these tools or systems developed externally, which could be customized for their local environment without investing the considerable resources [16, 17]. However, creating shareable CDS which could be deployed across different geographic areas requires prospective developers to 1) assess if recommended actions could be expressed logically in a formalized machine readable format and 2) identify existing differences in recommended actions for lead screening/management across guidelines [16, 18–21]. Significant discrepancies in recommendations across guidance sources could present a challenge for CDS authors. For instance, if one state recommends screening children based on zip code of residence while another recommends testing based on the location of a physician’s practice, this could result in over and under testing of children receiving care across state lines.

Prior work has examined differences in screening guidelines between states [22]; however, no work has explored the range of recommendations for outpatient management of elevated lead in pediatric populations. Ensuring children with elevated lead receive appropriate interventions is critical to addressing clinical consequences of lead toxicity, but also for identifying environmental sources. CDS offers a tool for improving lead screening and management adherence. To lay the groundwork for developing a shareable CDS, we reviewed publically available guidance for U.S. clinicians. Specifically, our goals were to 1) describe similarities and differences across recommendations for lead screening and management in children, and 2) assess feasibility of converting recommendation statements to CDS and customizing CDS for local guidance, and 3) identify factors that enable or pose significant barriers to the development of shareable CDS. As the CDC provides funding to states for lead screening and management programs, we also considered whether guidance from states receiving CDC funding provided more comprehensive screening and management recommendations [23].

## Methods

### Identification of Lead guidance

We evaluated lead guidance documents provided by all state public health departments and counties funded by the Centers for Disease Control (CDC) [23]. The CDC provides links to all state health departments’ lead pages regardless of whether they receive CDC funding and to the lead pages of counties’ health departments that receive funding. For this project, we grouped the District of Columbia with states for analysis. For CDC links that were broken, we used standard web searches to identify the guidance documents from state health departments.

A professional medical librarian performed a supplementary search for additional lead screening and management guidance within Embase, PubMed, ERIC, and CINAHL. Search strategies were customized for each database and included both controlled vocabulary terms and keywords. These keywords also informed the Google grey literature search. Search concepts included lead poisoning, elevated lead, management, treatment, therapy, guidelines, white papers, parameters, pathways, consensus, algorithm, and regulations. After reviewing documents to identify the breadth of recommendations contained in publically available guidance, we defined key elements for abstraction. These key elements included definitions of elevated lead levels (ELL), screening mandates, reporting requirements, clinical management, and follow-up schedule.

### Data abstraction

We designed a data abstraction tool using REDCap [24]. We abstracted information about guidance sources (e.g. publication year), elevated lead definition, screening, reporting, follow-up, and management recommendations. Levels of obligation for each clinical recommendation were determined using a deontic terminology framework [25]. The level of obligation classification system includes three categories (*must, should, or consider*) corresponding to the expectation for clinicians to carry out a given recommendation. For example, the expectation would be for clinicians to perform all *must* recommendations, while a *consider* recommendation might factor in patient preferences. Due to the rarity of *must* recommendations, we grouped *should* and *must* levels of obligation for analysis. Thus, all recommendations were mapped to two levels of provider obligation: *consider* and *should* [25].

For each recommendation we identified 1) intervention action, 2) threshold for physicians to consider (option “may”) performing the intervention, and 3) threshold for physicians being obligated (“should” or “must”) to perform the intervention. For follow-up lead testing, we identified test intervals between initial and confirmatory testing, early monitoring tests and late monitoring tests. Data were abstracted by one author and reviewed for accuracy by another member of the team.

### Analysis

We assessed whether receiving CDC funding or recent updating of guidance was associated with number of recommendation statements. We also investigated whether states having mandatory reporting or universal screening was associated with particular recommendations (e.g. recommendations for referral to early intervention).

Finally, we considered which discrete concepts across guidelines would require encoding to develop CDS. Concepts were categorized as decision variables or actions [26]. We selected appropriate standard clinical terminologies to represent each concept and identified barriers to identification of decision variables within the electronic health record (EHR) and executability of actions within the EHR.

All statistical tests were performed in Stata 13.1 (StataCorp LP). We tested for association between CDC funding, guidance publication date and cut-off values for elevated lead using Fisher’s exact test. We used the Mann-Whitney test to assess whether CDC funding was associated with number of distinct recommendations made by each guidance source.

## Results

Of 51 state public health departments, 48 provided at least minimal guidance on lead screening or management. For 3 states (Arkansas, North Dakota, Wyoming) we were unable to identify any form of publically available guidance for clinicians on lead screening or management.

Thirty-nine states and the District of Columbia receive funding from the CDC, while 11 states receive no funding. In addition, the CDC website linked to 11 city/county health department administered lead programs receiving CDC funding. Only 2 of 11 (New York City [27–29] and Philadelphia [30, 31]) included lead specific screening and management guidance and were included in our analysis. The other 9 counties received funding for activities related to decreasing lead burden (e.g., for patient evaluation services and/or home inspection services), but did not alter or supplement state provided clinical guidance and were therefore excluded from our analysis.

Supplemental searches identified additional lead screening and management guidance from 4 professional societies/government agencies: the American Academy of Pediatrics (AAP) [7, 32–34], the Center for Medicare and Medicaid Services (CMS) [35, 36], the CDC [37, 38], and the Pediatric Environmental Health Specialty Unit (PEHSU) [39]. In total, we identified 54 guidance sources published between 2008 and 2018 for inclusion: 48 state, 2 city/county, and 4 professional society/government agency (Table 1).

**Table 1:** Guidance Source Summary Data

Attribute	Sources examined (#)
Publication (year)^a^	54
• < 2012	• 7
• ≥ 2012	• 45
• No year^b^	• 2
Definition of ELL	54
• ≤ 3 μg/dL	• 1
• ≤ 5 μg/dL	• 43
• ≤ 10 μg/dL	• 10
Screening^c^	54
• Targeted	• 35
• Universal	• 18
• Not Addressed	• 1
Reporting (for states and District of Columbia only)	51
• Mandated All Results	• 43
• Mandated ELL Only	• 7
• Undefined	• 1
Clinical Management	54
• Provides Management Guidance	• 43
• No Management Guidance	• 11
Follow Up	54
• Provides Follow Up Guidance	• 40
• No Follow Up Guidance	• 14

^a^The AAP published a revised ELL in 2012, which we used as a cutoff for source evaluation

^b^Missouri and Nevada guidance sources lacked publication dates, but documents referenced in these sources indicate these policies were updated or reviewed during or after 2011 for Missouri and circa 2008 for Nevada

^c^Does not include NY city guidance as it does not differ from NY state guidance

### Definition of elevated Lead

All professional societies and government organizations defined elevated lead as ≥5 μg/dL, aligning with the CDC definition. However, definitions of ELL differed across states (Table 2). Of states providing guidance (*n* = 48), definitions of ELL ranged from 3 to 10 μg/dl, with the majority (55%, *n* = 37) using the ≥5 μg/dL cutoff. Notably, New York City specified a ≥ 5 μg/dL ELL threshold which differed from New York State’s definition of ≥10 μg/dL. Conversely, Philadelphia‘s threshold (ELL as ≥5 μg/dL) was consistent with Pennsylvania state guidance.

**Table 2:** Definitions of ELL for 50 US States and District of Columbia

ELL Definition (*N* = 51)	States
No level specified^a^ (3)	Arkansas [40], North Dakota [41], Wyoming [42]
Lead Level ≥ 3 μg/dL (1)	New Hampshire [43, 44]
Lead Level ≥ 5 μg/dL^b^ (37)	Alabama [45], Alaska [46], Arizona [47, 48], California [49, 50], Colorado [51, 52], Connecticut [53, 54], District of Columbia [55, 56], Georgia [57–59], Hawaii [60, 61], Idaho [62, 63], Illinois [64, 65], Iowa [66, 67], Kentucky [68, 69], Louisiana [70–72], Maine [73, 74], Maryland [75], Massachusetts [76–79], Michigan [80, 81], Minnesota [82, 83], Mississippi [84, 85], Montana [86], Nebraska [87, 88], New Mexico [89], North Carolina [90], Ohio [91, 92], Oklahoma [93, 94], Oregon [95, 96], Pennsylvania [97], Rhode Island [98, 99], South Carolina [100], South Dakota [101, 102], Tennessee [103], Texas [104, 105], Vermont [106, 107], Virginia [108–110], Washington [111–113], Wisconsin [114, 115]

^a^Lead is included as a ‘reportable disease’ but no ELL threshold is defined and no other guidance is given

^b^Montana does not provide screening guidance, but ELL is defined in the ‘reportable disease’ list when ≥5 μg/dL

^c^New York City defines ELL as ≥5 μg/dL, however the rest of New York State uses ELL as ≥10 μg/dL

Receiving CDC funding was not associated with adopting the CDC’s definition of ELL (≥ 5 μg/dL) or an even more stringent definition (e.g. > 3 μg/dL), (*p* = 1). However, older guidance (published prior to 2012) was associated with using the higher ELL threshold (*p* = 0.001). Only 4 states with guidance updated after 2012 continued to use the 10 μg/dL threshold at the time of our analysis (New Jersey, Delaware, Indiana, and Utah).

### Screening

Universal screening was recommended by 18 of 54 (33%) guidance sources (CMS for Medicaid recipients, PEHSU, 14 states, the District of Columbia, and the city of Philadelphia [the state of Pennsylvania recommends targeted screening]). Of 18 entities recommending universal screening, the majority (*n* = 15) recommended 2 screenings. The remaining 3 states (Delaware, Massachusetts, and Idaho) recommended 1, 3, and 5 screenings, respectively. With the exception of Montana (which only directs clinicians to the CDC for general guidance), all remaining guidance recommended targeted screening.

### Reporting requirements

We assessed reporting requirements for all 50 states and the District of Columbia. The majority (*n* = 43) mandated reporting of all lead results (regardless of level). For 7 states, reporting was mandated only when lead levels exceeded particular thresholds (greater than 2.3 μg/dL, 5 μg/dL, or 10 μg/dL). For one state (Nevada) we could not identify any reporting requirement after an extensive web search, a finding also reported by other investigators [22].

The interval of time permitted between testing and reporting results to a locality varied between states and by lead level. Some states had different reporting requirements for elevated lead vs. normal results. In some cases, reporting requirements within states differed by county. In some cases, states specified that levels above a certain μg/dL should be reported to a county health department instead of, or in addition to, the state health department. Many states already supported or required electronic result submission.

### Clinical Management

Of 54 guidance sources, 43 included recommendations for clinical management and care. We identified 17 distinct management recommendations (Table 3). On average, each guidance source indicated 5.5 identifiable distinct management recommendations (range 1–13). Many sources of guidance contained some similar recommendations (on average, a recommendation appeared in 13 guidance sources). Surprisingly, no recommendation was included across all guidance sources. The most common management recommendations were for in-person home inspection (*n* = 33), lead avoidance education (*n* = 32), and nutritional counseling (*n* = 25).

**Table 3:** Childhood Lead Management Recommendations

Management Recommendations (# of entities recommending an action in descending frequency)	Lead level thresholds to perform clinical actions (μg/dL)^a^ (# of entities recommending, cumulative by threshold)^b^								
	*≥ 0*	*≥ 3*	*≥ 5*	*≥ 10*	*≥ 15*	*≥ 20*	*≥ 25*	*≥ 45*	*≥ 70*
In person home inspection (33)	*–*	*–*	*6*	*18*	*26*	*33*	*	*	*
Lead avoidance education (32)	*17*	*18*	*31*	*32*	*	*	*	*	*
Nutrition counseling (25)	*4*	*5*	*22*	*25*	*	*	*	*	*
Iron testing (24)	*–*	*–*	*11*	*15*	*18*	*24*	*	*	*
Hospitalization (24)	*–*	*–*	*–*	*–*	*–*	*–*	*1*	*9*	*24*
Abdominal X-Ray (16)	*–*	*–*	*3*	*3*	*5*	*10*	*10*	*16*	*
Refer to social work (14)	*–*	*–*	*3*	*12*	*12*	*14*	*	*	*
Test family members (12)	*–*	*1*	*8*	*11*	*11*	*12*	*	*	*
Refer to Women, Infants, and Children (WIC) or nutrition (10)	*–*	*–*	*5*	*7*	*8*	*10*	*	*	*
In office exposure assessment (9)	*–*	*–*	*6*	*7*	*9*	*	*	*	*
Start iron or vitamin with iron (8)	*–*	*–*	*6*	*7*	*7*	*8*	*	*	*
Refer to early intervention (8)	*–*	*–*	*2*	*6*	*6*	*8*	*	*	*
File/Contact social services (8)	*–*	*–*	*2*	*5*	*5*	*6*	*6*	*8*	*
Refer to a lead clinic (7)	*–*	*–*	*1*	*1*	*1*	*3*	*3*	*7*	*
Phone exposure assessment (3)	*–*	*–*	*3*	*	*	*	*	*	*
Refer to medical specialty (2)	*–*	*–*	*1*	*1*	*1*	*1*	*1*	*2*	*
Add ELL to problem list (1)	*–*	*–*	*1*	*	*	*	*	*	*

^a^Some entities indicated a *consider* threshold and *should* threshold. When a single entity specified both, we tabulated based upon the *should* threshold

^b^A * indicates that all entities with this recommendation have been accounted for at a lower threshold

The lead level for clinicians to perform actions varied with regard to trigger thresholds and level of obligation (Table 3). For instance, of 33 guidance sources recommending an in-person home inspection, 6 recommended this intervention for all lead testing ≥5 μg/dL, while 7 only recommended an inspection for lead levels ≥20 μg/dL. One state recommended clinicians *consider* iron testing at ≥5 μg/dL and another state recommended iron testing *should* happen at ≥10 μg/dL. On a few occasions, states provided both a *consider* and *should* threshold for the same clinical management activity. For example, a guidance source recommended clinicians *consider* abdominal radiograph at ≥15 μg/dL, but *should* obtain one at levels ≥45 μg/dL [49]. Receiving CDC funding was not associated with higher number of distinct recommendations (*p* = 0.2).

### Follow-up testing

Forty (of 54) guidance sources addressed follow up lead testing (after initial screening) with confirmatory testing and/or monitoring. All states addressing elevated levels from capillary testing recommended confirmation with venous tests. Many states recommended confirmation for any result (capillary or venous) between 5 μg/dL and 19 μg/dL before making a diagnosis of elevated lead or initiating management. Intervals for confirmatory testing varied widely, ranging from 24 h to 3 months, depending on lead level and location of testing.

With regard to monitoring, we again found significant differences across recommendations depending on specimen type (capillary or venous), previous lead result, and lead result trends over time. Most guidance sources (82.5%, 33 of 40) offered different guidance for capillary compared to venous results. Sixty percent (24 of 40) of guidance sources included recommendations that changed based on lead level trends over time. Variability was high for lower lead levels (e.g. 5–20 μg/dl), but similar for patients with ELL ≥45 μg/dl. Some guidance statements provided age specific recommendations (older children (age ≥ 6) and adults). No guidance sources cited evidence to support follow up testing beyond a general reference to CDC or AAP guidance.

### Considerations for CDS development

We identified 20 discreet actions addressing screening (1), reporting (1), management (17), and follow-up (1). The logic for most recommendations was clear, unambiguous, and contained simple executable logic. All recommendations for lead screening and management were triggered by 5 patient and environment factors: patient age, office location (state), zip code of office, insurance type, and lead result history. These data are typically accessible within EHRs and sufficiently structured to permit use. Thus, we anticipate using parameters within the CDS Authoring Tool would allow for creation of practice specific rules (i.e. CDS reflecting local guidance). Furthermore, nearly all specified actions (*n* = 18) were directly related to the patient (and thus, amenable to implementation and adherence measurement). Only two recommendations did not directly involve the patient (recommendations for home testing and testing of family members). For these recommendations it was unclear who the actor should be (e.g. clinician or department of health representative).

Many recommendations (*n* = 10) required information that would need to be specified locally (e.g. fax number for early intervention, supplemental questions to ask on telephone assessment), but were otherwise executable. Thus, our analysis suggests current recommendations are amenable for conversion to CDS.

## Discussion

### Main findings of this study

We found numerous differences across guidance sources for screening and management of children with ELL. Eleven states do not use the CDC’s definition of elevated lead, and states offer variable guidance for what care children with ELL should receive. Only reporting requirements are relatively uniform across all states. These differences suggest any CDS intervention would require customization for local guidance, as many health systems span multiple localities. However, despite variability, recommendations were clear, concise, based upon a limited set of patient factors, and overwhelmingly executable suggesting they could translate well into effective CDS [116–118].

### What is already known on this topic

CDS has been previously developed to facilitate adherence to local guidance for lead screening and management. Our institution, for example, has incorporated initial lead screening within the well child care set as an optional order for relevant age groups. This basic CDS originally used only the most basic patient characteristics (age and insurance type) to determine for which patient it should be displayed. One local clinical site augmented this to support city guidance that differed from guidance across the rest of the network, and increased completed screening rates within the first 6 months from 73 to 81% and ordering rates from 85 to 95%. However, despite being effective, it has been difficult to share due to differences in applicable guidance, laboratory order options, and concerns about impact on workflow.

CDS, based upon a disseminated model, has proven an effective tool for addressing other pediatric public health problems. For example, CDS for immunizations has improved immunization rates [119–121]. While these immunization recommendations are the same across states, they are constantly updated. To address this, a shareable resource for immunization and CDS implementation was developed (CDSi), which allows local sites to develop and test immunization rules to ensure their patients are being provided the most appropriate care [122]. The team responsible for CDSi handles interpreting updates to immunization recommendation and encoding the computer logic necessary to produce the CDS. This allows organizations more time to focus on how best to incorporate these changes into workflow and to test changes without needing to reinvent the wheel. This disseminated CDS model demonstrates that shareable CDS initiatives can be used to address public health conditions.

### What this study adds

While geographic differences in lead exposure and competing priorities for resources may impact how localities decide to screen and manage childhood lead exposure [123, 124], the level of variability in screening and management guidelines is surprising. Differences with regard to universal versus targeted screening are understandable as local differences in environmental exposure result in difference risk-benefit ratios. However, state to state differences in what constitutes elevated lead and what management children should receive are problematic.

We found that receiving CDC funding was not associated with adopting current CDC definitions of elevated lead. However, older guidance (published before the CDC changed the ELL threshold) was more likely to use higher ELL thresholds. Comparison to prior work suggests more states have adopted ≥5 μg/dL threshold to define elevated lead since 2017, with only 10 states continuing to use 10 μg/dL threshold [22].

We found developing shareable CDS would be feasible despite this variability based on well written, actionable, and encodable recommendations. We anticipate healthcare organizations or health departments looking to sponsor CDS development for lead screening and management would need to overcome four challenges: 1) high volume of recommendations; 2) small differences between recommendations (i.e. handling the complex follow-up schedule); and 3) location specific concerns (e.g., regional risk factors for exposure) and 4) access to local resources (e.g. referral to local lead clinics and home inspection agencies). Cloud based or API driven solutions could work and have been used in the past for CDS [20, 21, 125]. However, new innovations in shareable CDS, including the Clinical Query Language [126], could better support CDS for lead screening and management through the embedded ‘parameters’ function, which was specifically developed to address this need for inter-organization variation [127].

### Limitations

As this project was aimed at describing the breadth of recommendations to inform CDS development, our efforts to identify recommendations was limited to web searches. Since we did not contact health departments directly, if websites were not regularly updated, our information may be inaccurate or outdated. However, these concerns are applicable to all web-search based research, and not specific to this project. As data abstraction from source documents was performed by a single abstractor, it is possible some recommendations were read or misinterpreted. However, this would have limited impact on our analysis, as a missed recommendation would likely exist in other guidance sources and have therefore have been evaluated for impact on shareable CDS development.

### Next steps

Given our findings and the large at-risk population, we believe developers should move quickly to develop and implement shareable CDS for lead screening and management. As a continuation of this effort, we are using the National Library of Medicine’s Value Set Authority Center to disseminate encodable representations of relevant clinical concepts identified during this project [128]. Also, our findings have been used to improve the alignment of current CDS at the Children’s Hospital of Philadelphia for lead screening with city, state, and national recommendations. We hope to demonstrate that adaptation of recommendations for lead screening and management can improve detection and management of lead in children and to study the spread of this intervention within and beyond the institution. Preliminary results suggest locally deployed CDS has significantly improved adherence to lead screening guidelines.

## Conclusions

Significant important differences across guidance for lead screening, reporting, management, and follow-up in the U.S. exists. These differences present both challenges and opportunities for CDS interventions. Although absence of a standardized definition of elevated lead and management recommendations poses challenges for the development of shareable CDS, this is balanced by well structured, actionable, and encodable recommendations. Although development of consensus regarding diagnosis of elevated lead and general management guidelines would be preferable, innovations in the field of shareable CDS can support local implementations when consensus among recommendation authors cannot be obtained.

## Data Availability

The datasets used and/or analyzed during the current study are available from the corresponding author on reasonable request. Please contact jmichel@ecri.org if you are interested in access to the data used in this study.

## Abbreviations

AAP: American Academy of Pediatrics
ADHD: Attention Deficit Hyperactivity Disorder
CDC: Center for Disease Control and Prevention
CDS: Clinical decision support
CHOP: Children’s Hospital of Philadelphia
CMS: Center for Medicare and Medicaid Services
EHR: Electronic Health Record
ELL: Elevated Lead Level
PEHSU: Pediatric Environmental Health Specialty Unit
